# Supplementary material for: Hybrid mRMR and multi-objective particle swarm feature selection methods and application to metabolomics of traditional Chinese medicine
Source: PeerJ Comput Sci. 2024 May 31;10:e2073. doi: 10.7717/peerj-cs.2073 (PMC11157565; doi:10.7717/peerj-cs.2073)
Supplement: Supplemental Information 3 [file peerj-cs-10-2073-s003.docx]

**Title：Hybrid mRMR and multi-objective particle swarm feature selection methods and application to metabolomics of traditional Chinese medicine**

Mengting Zhang^1^, Jianqiang Du^1,2,^*,Bin Nie^1,2^ Jigen Luo^1,2^,Ming Liu^1^,Yang Yuan^1^

^1^School of Computer Science, Jiangxi University of Traditional Chinese Medicine, 330004 Nanchang, Jiangxi, China

^2^Key Laboratory of Artificial Intelligence in Chinese Medicine, Jiangxi University of Traditional Chinese Medicine, 330004 Nanchang, Jiangxi, China

*Correspondence: jianqiang_du@163.com (Jianqiang Du)

***Supplementary Tables***

**Table S1：p-values obtained from hypothesis testing between MCMOPSO and the two comparison algorithms in the metabolomics dataset**

| DataSet | NSGAII-MCMOPSO | MOPSO-MCMOPSO |
| --- | --- | --- |
| Endo-y1 | 0.2607 | **0.0094** |
| Endo-y2 | 0.3361 | **0.0060** |
| Endo-y3 | **0.0001** | **0.0004** |
| Endo-y4 | 0.1254 | 0.1578 |
| Exo-y1 | **0.0186** | 0.6720 |
| Exo-y2 | **0.0030** | 0.096 |
| Exo-y3 | **0.0001** | **0.0005** |
| Exo-y4 | **0.0001** | 0.0671 |

The first column of the table represents the 8 datasets of metabolomics, and the second column is the p-value obtained from hypothesis testing of the results of NSGA-II and MCMOPSO groups, which is denoted as p1 in the article, and similarly the p-value obtained from MOPSO-MCMOPSO is denoted as p2.

**Table S2：p-values obtained from hypothesis testing between MCMOPSO and the two comparison algorithms in the UCI dataset**

| DataSet | NSGAII-MCMOPSO | MOPSO-MCMOPSO |
| --- | --- | --- |
| BlogTe1 | **0.0133** | 0.1630 |
| BlogTe2 | 0.1049 | 0.2216 |
| BlogTe3 | **0.0478** | 0.1758 |
| BlogTe4 | **3.04E-06** | 0.3627 |
| BlogTe5 | 0.4569 | 0.5021 |
| BlogTe6 | 0.2601 | 0.1935 |
| BlogTe7 | **8.46E-09** | **0.0001** |
| BlogTe8 | **1.21E-07** | **0.0212** |
| BlogTe9 | 0.6771 | **0.0171** |
| BlogTe10 | **0.0116** | **0.0310** |

The first column of the table represents the 10 datasets of UCI, and the second column is the p-value obtained from hypothesis testing of the results of NSGA-II and MCMOPSO groups, which is denoted as p1 in the article, and similarly the p-value obtained from MOPSO-MCMOPSO is denoted as p2.

**Table S3：Comparison of R-square and MAE Results for One-Stage mRMR Algorithm and Other Comparative Algorithms on Metabolomic Dataset**

|  | **R^2** | | | | | **MAE** | | | | | |
| --- | --- | --- | --- | --- | --- | --- | --- | --- | --- | --- | --- |
| **DataSet** | **fu11_set** | **mRMR** | **MIC** | **Pearson** | **Spearman** | **fu11_set** | **mRMR** | **MIC** | **Pearson** | **Spearman** |  |
| Endo-y1 | -0.1181 | **0.5809** | 0.0090 | 0.1137 | 0.1023 | 559.7119 | **348.1721** | 507.0500 | 458.5555 | 486.7036 |  |
| Endo-y2 | -0.3651 | **0.6704** | -0.0630 | -0.1557 | 0.0873 | 22.1788 | **11.4727** | 19.8968 | 19.4281 | 18.0039 |  |
| Endo-y3 | 0.2532 | **0.6611** | 0.3121 | 0.3454 | 0.2746 | 4.0441 | **2.8775** | 4.0487 | 3.8494 | 4.0393 |  |
| Endo-y4 | 0.0821 | **0.5009** | 0.3033 | 0.3806 | 0.3255 | 8.9873 | **6.5481** | 7.8969 | 7.3776 | 8.0534 |  |
| Exo-y1 | 0.6191 | 0.8190 | 0.8029 | **0.8421** | 0.7733 | 276.3393 | 165.0824 | 183.9974 | **153.6420** | 195.6676 |  |
| Exo-y2 | -1.3923 | -1.4036 | **0.9698** | -1.39233 | -0.5633 | 12.8306 | 12.8538 | **12.5400** | 12.83057 | 11.9899 |  |
| Exo-y3 | 0.6039 | **0.7501** | 0.5938 | 0.6039 | 0.6039 | 2.8719 | **2.0937** | 2.8899 | 2.8719 | 2.8719 |  |
| Exo-y4 | -0.3201 | **0.0700** | -0.1176 | -0.3201 | -0.3201 | 3.0504 | **2.6716** | 2.7611 | 3.0504 | 3.0504 |  |

The first row is the two evaluation metrics are: r-squared and MAE. The table shows the r-squared sum MAEs of the output features of mRMR and the three comparison algorithms for the eight datasets of metabolomics, compared with the r-squared sum MAEs of the full set. Among them, the numbers marked in red are the ones with the best results.

**Table S4：Comparison of R-square and MAE Results for One-Stage mRMR Algorithm and Other Comparative Algorithms on UCI Dataset**

|  | | **R^2** | | | | | **MAE** | | | | | |
| --- | --- | --- | --- | --- | --- | --- | --- | --- | --- | --- | --- | --- |
| **DataSet** | **fu11_set** | | **mRMR** | **MIC** | **Pearson** | **Spearman** | **fu11_set** | **mRMR** | **MIC** | **Pearson** | **Spearman** |  |
| BlogTe1 | 0.1743 | | 0.3311 | **0.5626** | 0.4663 | 0.4187 | 10.9571 | 9.3398 | **8.2193** | 9.7748 | 9.9025 |  |
| BlogTe2 | 0.1915 | | 0.4273 | 0.4861 | 0.3180 | **0.5012** | 9.0325 | 7.1527 | 7.0898 | 7.3920 | **6.5557** |  |
| BlogTe3 | -4.0984 | | 0.2845 | 0.2299 | 0.3042 | **0.3497** | 20.0693 | 10.9354 | 10.6507 | 10.5102 | **9.6664** |  |
| BlogTe4 | -5.0274 | | -0.6102 | -0.4118 | **-0.3167** | -0.6484 | 7.0723 | 5.6802 | 5.3362 | **4.8346** | 5.3698 |  |
| BlogTe5 | 0.5166 | | 0.8182 | 0.7995 | 0.7701 | **0.8547** | 8.9576 | 6.7633 | 6.4929 | 6.1980 | **6.0405** |  |
| BlogTe6 | 0.4385 | | 0.5128 | 0.5787 | **0.6532** | 0.4535 | 6.7065 | **4.8972** | 5.6182 | 5.1607 | 6.7184 |  |
| BlogTe7 | 0.14072 | | 0.1407 | 0.3300 | **0.3546** | 0.3171 | 4.9435 | 4.9435 | 4.9180 | **4.1255** | 4.6857 |  |
| BlogTe8 | -0.9002 | | -0.1731 | **0.0204** | -0.1871 | -0.9002 | 18.2467 | 15.9966 | **11.9452** | 15.7116 | 18.2467 |  |
| BlogTe9 | -0.1756 | | -0.0559 | -0.0637 | -0.0154 | **-0.0095** | 11.4928 | 11.0438 | **9.4501** | 11.2066 | 10.7230 |  |
| BlogTe10 | 0.3050 | | 0.5002 | 0.4011 | **0.5100** | 0.3252 | 8.8399 | 7.0032 | **6.3393** | 6.8869 | 8.9682 |  |

The first row is the two evaluation metrics are: r-squared and MAE. The table shows the r-squared sum MAEs of the output features of mRMR and the three comparison algorithms for the 10 datasets of UCI, compared with the r-squared sum MAEs of the full set. Among them, the numbers marked in red are the ones with the best results.

**Table S5：Comparison of R-square and MAE results between the MCMOPSO algorithm and other comparative algorithms on the metabolomic dataset in Phase 2**

|  | | **R^2** | | | **MAE** | | | |
| --- | --- | --- | --- | --- | --- | --- | --- | --- |
| **DataSet** | **MCMOPSO** | | **NSGA-II** | **MOPSO** | | **MCMOPSO** | **NSGA-II** | **MOPSO** |
| Endo-y1 | **0.7257** | | 0.714922 | 0.716857 | | **288.6019427** | 291.4226 | 290.3135 |
| Endo-y2 | **0.743982635** | | 0.737276 | 0.732571 | | 10.02967893 | 10.20625 | **10.1666** |
| Endo-y3 | **0.761185007** | | 0.748264 | 0.742535 | | **2.289880421** | 2.37478 | 2.391651 |
| Endo-y4 | **0.686941321** | | 0.672126 | 0.671605 | | **4.982693102** | 5.051008 | 5.085118 |
| Exo-y1 | 0.921918788 | | 0.9223 | **0.9234** | | 120.8093657 | 119.2378 | **118.3406** |
| Exo-y2 | **0.184870022** | | 0.178639 | 0.148984 | | **8.712371574** | 8.741666 | 8.887751 |
| Exo-y3 | **0.850819928** | | 0.827812 | 0.830245 | | **1.624974466** | 1.718673 | 1.714707 |
| Exo-y4 | **0.409965919** | | 0.36348 | 0.401481 | | 1.967763295 | 2.014016 | **1.953653** |

The table shows the comparison of the R-squared and MAE results obtained by MCMOPSO in the second stage with the NSGA-II algorithm and the MOPSO algorithm. Therein, the first row shows the two evaluation metrics: the R-squared and the MAE. the first column shows the 8 datasets of metabolomics and the other columns show the values of the algorithm names corresponding to the corresponding metrics.

**Table S6：Comparison of R-square and MAE results between the MCMOPSO algorithm and other comparative algorithms on the UCI dataset in Phase 2**

|  | **R^2** | | | | **MAE** | | | |
| --- | --- | --- | --- | --- | --- | --- | --- | --- |
| **DataSet** | | **MCMOPSO** | **NSGA-II** | **MOPSO** | **MCMOPSO** | **NSGA-II** | **MOPSO** |  |
| BlogTe1 | | **0.900973004** | 0.745143 | 0.813862 | **6.47120136** | 6.770505 | 7.062665 |  |
| BlogTe2 | | **0.854360895** | 0.684603 | 0.821888 | **4.024588155** | 5.197601 | 4.412731 |  |
| BlogTe3 | | **0.907383645** | 0.822369 | 0.036518 | **4.837829258** | 5.68264 | 10.22531 |  |
| BlogTe4 | | 0.086449649 | 0.106412 | **0.092157** | **3.952645802** | 4.002014 | 4.039169 |  |
| BlogTe5 | | **0.924442034** | 0.922997 | 0.914008 | **3.563438086** | 3.91938 | 3.837502 |  |
| BlogTe6 | | 0.901406057 | **0.908121** | 0.89058 | 3.61825215 | **3.561007** | 3.682778 |  |
| BlogTe7 | | 0.415784051 | **0.534503** | 0.468942 | 4.597324403 | **4.073159** | 4.470072 |  |
| BlogTe8 | | 0.221172612 | **0.293554** | 0.269415 | 13.70682168 | **11.29582** | 13.51557 |  |
| BlogTe9 | | 0.57207714 | 0.557503 | **0.576487** | **8.391738745** | 8.507358 | 8.423208 |  |
| BlogTe10 | | **0.815493745** | 0.804504 | 0.812436 | 5.112049815 | **5.104849** | 5.150851 |  |

The table shows the comparison of the R-squared and MAE results obtained by MCMOPSO in the second stage with the NSGA-II algorithm and the MOPSO algorithm. Therein, the first row shows the two evaluation metrics: the R-squared and the MAE. the first column shows the 10 datasets of UCI and the other columns show the values of the algorithm names corresponding to the corresponding metrics.
